# Development and validation of a novel prognostic prediction system based on GLIM-defined malnutrition for colorectal cancer patients post-radical surgery

**DOI:** 10.3389/fnut.2024.1425317

**Published:** 2024-10-22

**Authors:** Xialin Yan, Junchang Zhu, Junqi Wang, Yingjie Lu, Xingzhao Ye, Xiangwei Sun, Haojie Jiang, Zongze Li, Chenhao He, Wenbo Zhai, Qiantong Dong, Weizhe Chen, Zhen Yu, Yifei Pan, Dongdong Huang

**Affiliations:** ^1^Department of Colorectal and Anal Surgery, The First Affiliated Hospital of Wenzhou Medical University, Wenzhou, China; ^2^Department of Gastrointestinal Surgery, The First Affiliated Hospital of Wenzhou Medical University, Wenzhou, China; ^3^Department of Gastrointestinal Surgery, Shanghai Tenth People’s Hospital, School of Medicine, Tongji University, Shanghai, China

**Keywords:** global leadership initiative on malnutrition, clinical nutrition, colorectal cancer, nomogram, clinical outcomes

## Abstract

**Background:**

Malnutrition often occurs in patients with colorectal cancer. This study aims to develop a predictive model based on GLIM criteria for patients with colorectal cancer who underwent radical surgery.

**Methods:**

From December 2015 to May 2021, patients with colorectal cancer who underwent radical surgery at our center were recruited for this study. We prospectively collected data on GLIM-defined malnutrition and other clinicopathological characteristics. Using Cox regeneration, we developed a novel nomogram for prognostic prediction, which was validated and compared to traditional nutritional factors for predictive accuracy.

**Results:**

Among the 983 patients enrolled in this study, malnutrition was identified in 233 (23.70%) patients. Multivariate analysis indicated that GLIM-defined malnutrition is the independent risk factor for overall survival (HR = 1.793, 95% CI = 1.390–2.313 for moderate malnutrition and HR = 3.485, 95% CI = 2.087–5.818 for severe malnutrition). The novel nomogram based on the GLIM criteria demonstrated a better performance than existing criteria, with AUC of 0.729, 0.703, and 0.683 for 1-year, 3-year, and 5-year OS, respectively, in the validation cohort. In addition, the risk score determined by this system exhibited significantly poorer short-term and long-term clinical outcomes in high-risk groups in both malnourished and well-nourished patients.

**Conclusion:**

Combining handgrip strength, serum albumin level, and TNM stage would help improve the predictive effect of GLIM criteria for colorectal cancer patients post-radical surgery and benefit the individual prognostic prediction of colorectal cancer.

## 1 Introduction

With the third cancer incidence and second cancer-related mortality, colorectal cancer (CRC) remains the rising burden worldwide (1). Prediction and assessment of the clinical outcomes after radical colectomy are still the major concerns of surgeons. Like other severe diseases in the intestinal tract, malnutritional status is often witnessed in patients with CRC. Fasting before or after surgical treatment, hypermetabolism, symptoms of nausea or vomiting, and anemia or obstruction caused by CRC, especially in the advanced stage, are also blamed for malnutrition (2, 3). Thus, malnutrition is also regarded as an indicator of the development of CRC disease.

Malnutrition is now recognized as significantly related to poorer physical health before surgery and has an impact on both the short- and long-term prognosis of cancer patients (4, 5). Recently, the Global Leadership Initiative on Malnutrition (GLIM), consisting of three phenotypic criteria and two etiologic criteria, was published as a global consensus for the diagnosis of malnutrition (2, 6, 7), which has exhibited its advantages for prognostic assessment in gastric cancer in our previous studies (8, 9). For phenotypic criteria of GLIM, reduced muscle mass is assessed by a variety of methods, including computerized tomography (CT) scanning, bioelectrical impedance analysis (BIA), dual-energy X-ray absorptiometry (DXA), and anthropometry, which would correspond to various incidences of reduced muscle mass and GLIM-defined malnutrition (10–12). CT scanning is regarded as the standardized radiological assessment of skeletal muscle mass for its accuracy even in special conditions, such as excess adiposity or edema, and has been reported to evaluate reduced muscle mass for GLIM (10, 13). In addition, skeletal muscle function, while not a surrogate measurement of reduced muscle mass in GLIM, is an important component of sarcopenia to complete nutrition assessment after malnutrition is confirmed (11). However, with the dynamic alternation in nutritional status during the development of CRC disease, making a precise prediction on long-term prognosis based on the nutritional status of each patient remains hard and complicated.

Recent studies have introduced the combination of GLIM-defined malnutrition and other parameters, such as surgical factors, to achieve better predictive effectiveness (9, 14). In the present study, clinicopathological characteristics and data on skeletal muscle quantity and function, including CT-determined skeletal muscle mass and handgrip strength, were collected prospectively. Then, based on GLIM-defined malnutrition and other screened elements related to nutritional status and skeletal muscle function, we aimed to develop a simple but precise tool for prognostic prediction in CRC patients, which may guide nutritional support in clinical practice.

## 2 Materials and methods

### 2.1 Patients

From December 2015 to May 2021, consecutive patients with stages I–III colorectal cancer were recruited in this study based on a prospective cohort. The inclusion criteria were as follows: (1) age ranging from 18 to 80 years old; (2) diagnosis of primary colorectal adenocarcinoma confirmed pathologically, having undergone radical colectomy or proctectomy; and (3) availability of data for risk screening or diagnosis of malnutrition. Exclusion criteria included: (1) confirmed evidence of distant metastasis or receipt of palliative surgery; (2) emergency surgery; and (3) inability to assess skeletal muscle mass and function. Written informed consent forms were obtained from all the patients recruited in this study, and study methodologies were approved by the Ethics Committee of the First Affiliated Hospital of Wenzhou Medical University (KY2023-R055).

### 2.2 Collection of clinical data

The clinical data of participants were collected prospectively, which includes: (1) demographic features, such as gender, age, body mass index (BMI), American Society of Anesthesiologists (ASA) grade, Charlson Comorbidity Index (CCI), history of previous abdominal surgery, concentration of preoperative serum albumin (Alb), and hemoglobin (Hb); (2) tumor-related details, including tumor location and TNM stage; (3) surgical information, such as operative time, number of harvested lymph nodes, and stoma; (4) data for evaluating nutritional status, including weight loss, decreased food intake, and reduced muscle mass determined by skeletal muscle index (SMI) from L3 level of abdominal CT scanning based on our previous research (8) and parameters related to muscle function such as grip strength and gait speed; (5) clinical outcomes, such as postoperative complications (severe complication was defined as grade ≥ 3 according to the Calvien–Dindo scoring system), length of hospital stay, overall survival (OS), and disease-free survival (DFS).

### 2.3 Follow-up

The routine follow-up by telephone interviews or outpatient visits was carried out 1 month after discharge, every 3 months for the first 2 years, and every half year thereafter. The dates of death and recurrence were recorded to determine OS and DFS. The median follow-up time in this study was 3.53 years.

### 2.4 Diagnosis of GLIM-defined malnutrition

Malnutrition was diagnosed following GLIM criteria via the two-step approach, according to a previous study (9). Briefly, patients were first assessed by Nutritional Risk Screening 2002 (NRS-2002) to identify nutritional risk. Patients with a score of NRS-2002 ≥ 3 proceeded to the next step of malnutritional diagnosis, which consists of three phenotypic criteria (non-volitional weight loss, low BMI, and reduced muscle mass) and two etiologic criteria (reduced food intake or assimilation and disease burden or inflammation). In detail, patients with colorectal cancer met the disease burden based on the etiologic criteria for malnutrition. Low BMI was defined as BMI < 18.5 kg/m^2^, if age < 70 or BMI < 20 kg/m^2^, if age > 70, based on the cutoff value for the Asian population (15), and reduced muscle was determined by the SMI as reported in our previous research (9). The patients who met at least one out of the phenotypic criterion and one etiologic criterion were diagnosed with GLIM-defined malnutrition, which was graded according to the cutoff values recommended for the Asian population (BMI < 17.0 kg/m^2^, if age < 70 years or BMI > 17.8 kg/m^2^, if age < 70 years) (16).

### 2.5 Statistical analysis

Quantitative variables were expressed as mean ± standard deviations (SD) and analyzed using Student’s *t*-test if following a normal distribution, or the data were expressed as medians with interquartile ranges (IQR) and conducted using the Mann–Whitney *U*-test. Categorical variables were expressed as numbers with proportions (n (%)) and analyzed using the chi-squared or Fisher’s correction if necessary. Kaplan–Meier curves were conducted to describe the difference of OS and DFS between the groups. Clinical characters of the patients were screened through univariate analysis and following multivariate analysis for the factor with *p* < 0.05 to formulate the nomogram aiming to predict long-term survival. Receiver operating characteristic curve (AUC) and decision curve analysis (DCA) were performed to evaluate the accuracy and discriminative ability of the nomogram model and other models. All the statistical analyses were conducted using SPSS software (version 26.0) and R software (version 4.1.0), with RStudio (version 1.2.5033). A two-tailed *p* < 0.05 was regarded as a significant difference.

## 3 Results

### 3.1 Baseline features

A total of 983 patients with stages I–III colorectal cancer who underwent radical surgery were recruited in this study between December 2015 and May 2021. These patients were then randomly allocated with a ratio of 2:1 to obtain the training (n = 656) and validation cohort (n = 327). The demographic characters and clinical characters are shown in Table 1. In both training and validation cohorts, patients with GLIM-defined malnutrition were significantly older (*p* < 0.001 and *p* = 0.004, respectively), had lower BMI (*p* < 0.001 and *p* < 0.001, respectively), lower preoperative serum albumin (Alb) (*p* < 0.001 and *p* < 0.001, respectively), and hemoglobin (Hb) (*p* < 0.001 and *p* < 0.001, respectively) levels. Compared to well-nourished patients, significantly lower SMI (*p* < 0.001 and *p* < 0.001, respectively), poorer performance of handgrip strength (*p* < 0.001 and *p* < 0.001, respectively), and lower gait speed (*p* < 0.001 and *p* < 0.072, respectively) were also found in the malnutrition group in both cohorts.

**Table 1:** Comparison of the clinical characteristics of training and validation cohorts.

	Training cohort (*n* = 656)			Validation cohort (327)		
	GLIM diagnosis			GLIM diagnosis		
	Non-malnutrition (*n* = 493)	Malnutrition (*n* = 163)	*p-*value	Non-malnutrition (*n* = 257)	Malnutrition (*n* = 70)	*p-*value
Age, median (IQR), year	63 (15)	70 (16)	<0.001	64 (14)	66 (14)	0.004
Sex, *n* (%)			0.335			0.092
Male	310 (62.88)	96 (58.90)		178 (69.26)	41 (58.57)	
Female	183 (37.12)	67 (41.10)		79 (30.74)	29 (41.43)	
BMI, median (IQR), (kg/m^2^)	23.43 (3.76)	20.45 (3.93)	<0.001	23.37 (3.90)	20.13 (3.94)	<0.001
ASA grade, *n* (%)			0.119			0.496
I	150 (30.43)	40 (24.54)		73 (28.40)	26 (37.14)	
II	295 (59.84)	99 (60.74)		158 (61.49)	38 (54.29)	
III	48 (9.74)	24 (14.72)		26 (10.11)	6 (8.57)	
TNM stage, *n* (%)			0.237			0.019
I	126 (25.56)	31 (19.02)		59 (22.96)	8 (11.43)	
II	205 (41.58)	74 (45.40)		112 (43.58)	27 (38.57)	
III	162 (32.86)	58 (35.58)		86 (33.46)	35 (50.00)	
Tumor location, *n* (%)			0.479			0.210
Colon	290 (58.82)	101 (61.96)		140 (54.47)	44 (63.86)	
Rectum	203 (41.18)	62 (38.04)		117 (45.53)	26 (37.14)	
Charlson comorbidity index, *n* (%)			0.884			0.997
0	235 (47.67)	79 (48.47)		128 (49.81)	35 (50.00)	
1	173 (35.09)	54 (33.13)		84 (32.68)	23 (32.86)	
≥2	85 (17.24)	30 (18.40)		45 (17.51)	12 (17.14)	
Previous abdominal surgery, *n* (%)			0.018			0.744
Yes	93 (18.86)	45 (27.61)		47 (18.29)	14 (20.00)	
No	400 (81.14)	118 (72.39)		210 (81.71)	56 (80.00)	
SMI, median (IQR) (kg/m^2^)	44.66 (11.16)	38.61 (9.13)	<0.001	44.79 (11.55)	39.43 (10.16)	<0.001
Hand grip strength, median (IQR) (kg)	26.60 (14.50)	22.70 (14.30)	<0.001	27.90 (12.50)	22.10 (12.30)	<0.001
Gait speed, median (IQR) (m/s)	0.91 (0.24)	0.83 (0.26)	<0.001	0.91 (0.27)	0.85 (0.28)	0.072
Hemoglobin, median (IQR) (g/L)	128.00 (30.00)	119.00 (27.00)	0.001	129.00 (32.00)	115.00 (34.00)	0.001
Serum albumin, median (IQR) (g/L)	38.70 (5.30)	37.60 (5.60)	0.001	38.50 (5.30)	37.60 (7.30)	0.001

The percentage of patients who were malnourished and met a single GLIM criterion in each cohort is presented in Supplementary Table 1. Briefly, in the current cohort, 233 (23.70%) patients were identified as having malnutrition based on GLIM criteria, among which 24 patients were graded as having severe malnutrition. Low BMI was identified in 48 (7.32%) patients in the training cohort and 24 (7.34%) patients in the validation cohort. Reduced muscle mass was found in 160 (24.39%) and 79 (24.16%) patients in the training and validation cohort, respectively.

### 3.2 GLIM-defined malnutrition and clinical outcomes

As shown in Table 2, postoperative complications occurred in 29.18% (68/233) of the patients with GLIM-defined malnutrition, and a significantly lower incidence of postoperative complications (22.00%) was found in the non-malnutrition group (*p* = 0.024). The lower incidence of severe complications (3.20 vs. 5.15%) and shorter postoperative hospital stay (11.20 ± 6.47 days vs. 13.91 ± 6.60 days) were also identified in the non-malnutrition group, but not significantly different. Similar results were also found in the training and validation cohorts. Moreover, stomas tend to be more frequently performed for patients with malnutrition (4.80 vs. 9.44%, *p* = 0.009). This finding supports the previous studies that malnutrition is one of the risk factors for anastomotic leak, so instead of primary anastomosis, a stoma was performed to lessen the consequences of anastomotic leak and other potential complications (17, 18).

**Table 2:** Effects of GLIM-defined malnutrition on short-term clinical outcomes.

	Total			Training cohort			Validation cohort		
	GLIM diagnosis			GLIM diagnosis			GLIM diagnosis		
	Non-malnutrition (*n* = 750)	Malnutrition (*n* = 233)	*p-*value	Non-malnutrition (*n* = 493)	Malnutrition (*n* = 163)	*p-*value	Non-malnutrition (*n* = 257)	Malnutrition (*n* = 70)	*p-*value
Total complications, *n* (%)	165 (22.00)	68 (29.18)	0.024	111 (22.52)	47 (28.83)	0.102	54 (21.01)	21 (30.00)	0.08
Severe complications, *n* (%)	24 (3.20)	12 (5.15)	0.166	16 (3.25)	9 (5.52)	0.188	8 (4.67)	3 (4.29)	0.914
Postoperative hospital stay, (d)	11 (6)	12 (5)	0.14	11 (5)	12 (5)	0.625	11 (5)	13 (7)	0.041
Operative time, median (IQR) (min)	180.00 (76.00)	171.00 (79.00)	0.15	171.00 (70.00)	167.00 (75.00)	0.061	179.00 (72.00)	172.00 (74.00)	0.682
Stoma, *n* (%)	36 (4.80)	22 (9.44)	0.009	23 (4.67)	14.00 (8.59)	0.06	13 (5.06)	8.00 (11.43)	0.001
Number of harvested Lymph nodes, median (IQR)	18.00 (3.00)	18.00 (2.00)	0.072	17.00 (3.00)	18.00 (3.00)	0.243	18.00 (3.00)	18.00 (2.00)	0.119
Readmissions within 30 d of discharge, *n* (%)	15 (2.00)	9 (3.86)	0.108	9 (1.83)	6 (3.68)	0.284	6 (2.23)	3 (4.29)	0.637

**Figure 1:**
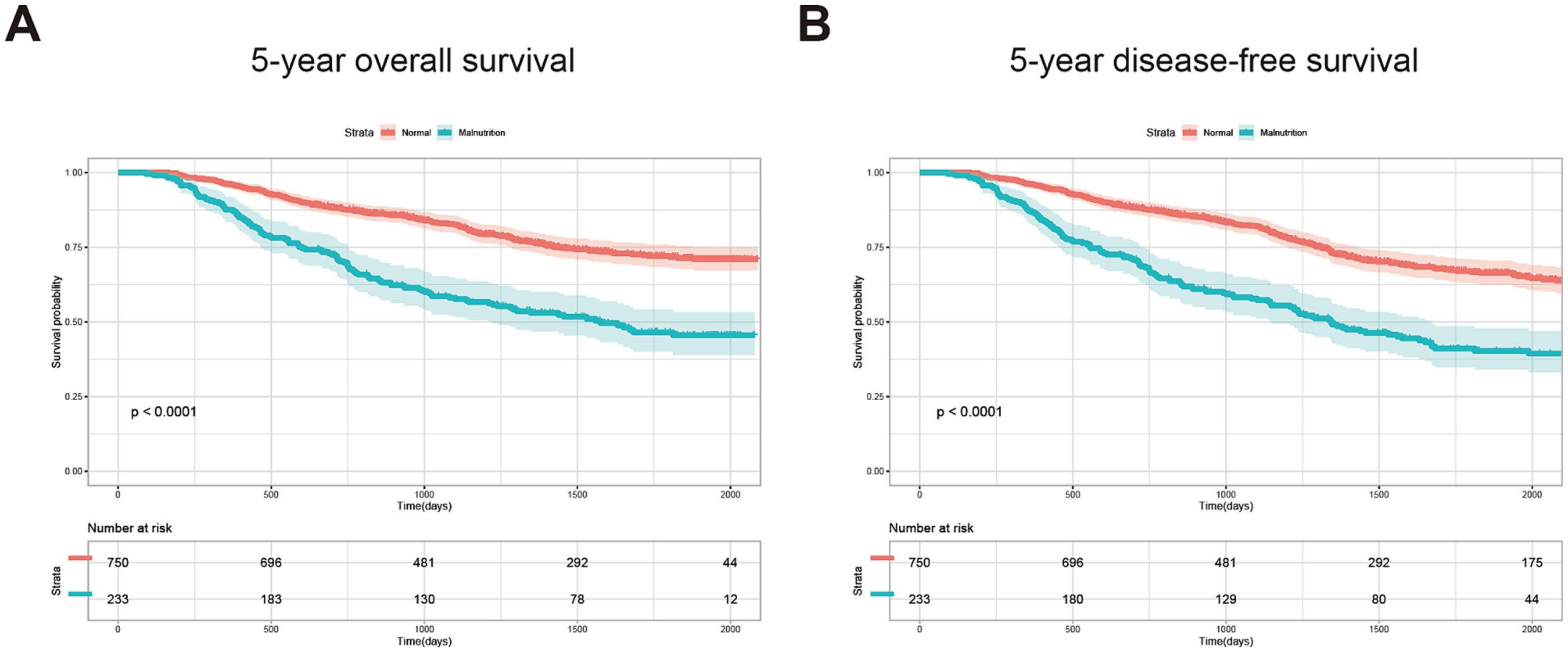
Kaplan–Meier curves of 5-year OS **(A)** and 5-year DFS **(B)** for the patients with or without GLIM-defined malnutrition.

For long-term prognosis, 238 patients died during the follow-up period. We then performed the K–M plot of these patients. The survival curve indicated that the patients with malnutrition demonstrated significantly poorer 5-year OS and 5-year DFS, which are shown in Figure 1, and the survival was even poorer for the patients with severe malnutrition (Supplementary Figure 1). Clinical factors that may affect long-term survival were analyzed using univariate and multivariate Cox regression (Table 3). In multivariate analysis, except for GLIM-defined malnutrition (*p* < 0.001), age (*p* = 0.001), TNM stage (*p* = 0.001), handgrip strength (*p* = 0.028), and serum albumin level (*p* = 0.042) were identified as independent prognostic factors for OS in patients who underwent radical colectomy or proctectomy.

**Table 3:** Univariate and multivariate analyses for 5-year overall survival.

Factors	Univariate analysis	*p*-value	Multivariate analysis	*p*-value
	HR (95% CI)		HR (95% CI)	
Age	1.036 (1.024–1.049)	<0.001	1.022 (1.009–1.034)	0.001
Sex, male	0.841 (0.665–1.062)	0.145		
BMI	0.956 (0.919–0.993)	0.021		
ASA grade		0.022		
I	1 (Reference)			
II	1.183 (0.909–1.539)	0.212		
III	1.712 (1.169–2.507)	0.006		
TNM stage		<0.001		<0.001
I	1 (Reference)			
II	1.419 (1.009–1.995)	0.044	1.258 (0.891–1.775)	
III	2.066 (1.475–2.893)	<0.001	1.896 (1.349–2.664)	<0.001
Tumor location		0.284		
Colon	1 (Reference)			
Rectum	1.134 (0.901–1.428)			
NRS-2002 ≥ 3	1.931 (1.534–2.429)	<0.001		
GLIM malnutrition		<0.001		<0.001
Normal	1 (Reference)			
Moderate	2.267 (1.772–2.900)	<0.001	1.793 (1.390–2.313)	<0.001
Severe	4.869 (2.951–8.034)	<0.001	3.485 (2.087–5.818)	<0.001
Charlson comorbidity index		0.094		
0	1 (Reference)			
1	1.255 (0.971–1.621)	0.083		
≥2	1.346 (0.983–1.845)	0.064		
Previous abdominal surgery, yes	1.190 (0.903–1.568)	0.216		
SMI	0.979 (0.967–0.992)	0.001		
Handgrip strength	0.964 (0.950–0.977)	<0.001	0.985 (0.972–0.998)	0.028
Gait speed	0.967 (0.955–0.979)	<0.001		
Hemoglobin	0.992 (0.987–0.997)	0.001		
Serum albumin	0.942 (0.918–0.967)	<0.001	0.971 (0.943–0.999)	0.042

### 3.3 Establishment and validation of the prognostic nomogram

**Figure 2:**
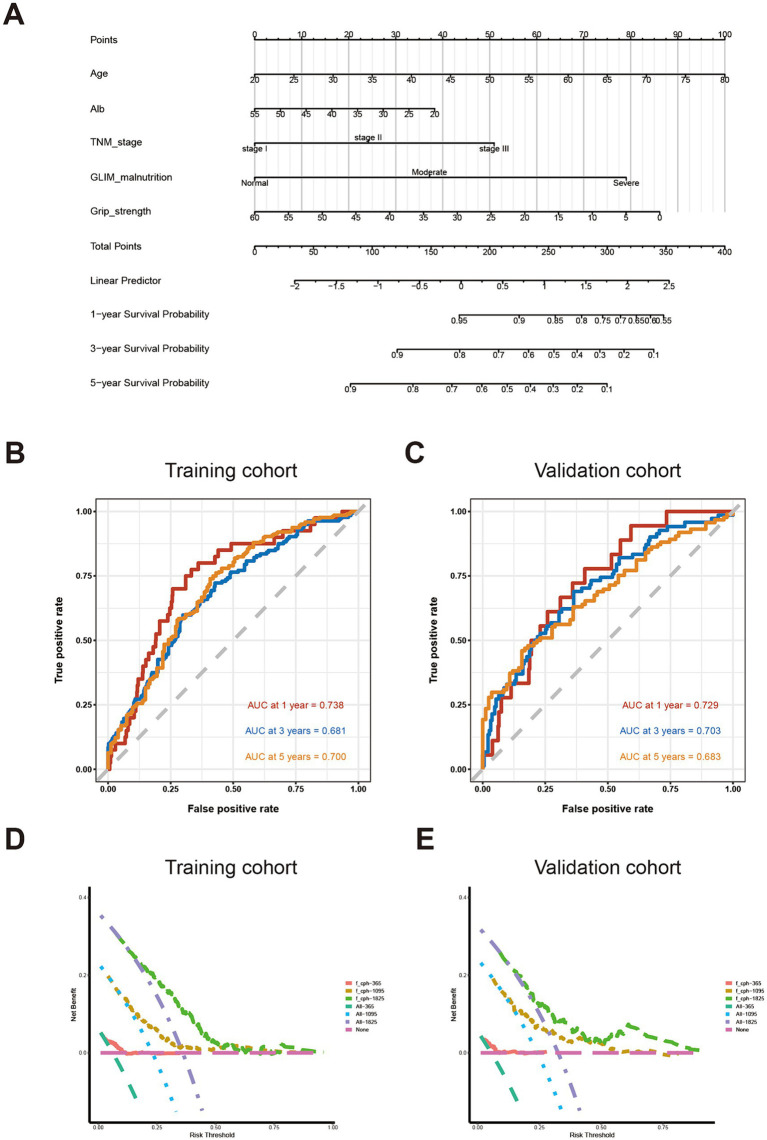
Establishment and validation of predictive system based on identified factors. **(A)** Nomogram based on age, serum album level, TNM staging, GLIM-defined malnutrition, and handgrip strength **(B)** AUC for survival prediction in training cohort; **(C)** AUC for survival prediction in validation cohort; **(D)** DCA for survival prediction in training cohort; **(E)** DCA for survival prediction in the validation cohort.

Based on the factors identified using multivariate Cox regression analysis, clinical factors, including age, serum album level, pathological TNM stage, GLIM-defined nutritional status, and grip strength, were integrated for developing the novel nomogram to predict OS (Figure 2A). A point scale was used to obtain the score of each factor and the probability of OS. The OS was defined by the total score displayed on the total score scale. The nomogram yield AUC values were 0.738 for 1-year OS, 0.681 for 3-year OS, and 0.700 for 5-year OS in the training cohort (Figure 2B), and 0.729 for 1-year OS, 0.703 for 3-year OS, and 0.683 for 5-year OS in the validation cohort, respectively (Figure 2C). The decision curve analysis (DCA) curve of the nomogram in the training and validation cohorts is presented in Figures 2D,E, respectively.

In order to further evaluate the prognostic effect of the novel nomogram system based on nutrition-related factors, we compared its AUC value with those common clinical factors used for screening malnutrition and predicting long-term prognosis for patients with CRC (19–21). Therefore, BMI, Alb, Hb, etc., served as an indicators to predict OS. As shown in Supplementary Figure 2, the AUC values of those predictors mostly ranged from 0.4 to 0.6 for 1-year, 3-year, and 5-year OS in the training or validation cohort, which were much lower than the AUC values of our novel nomogram system.

### 3.4 Risk grading of the individual predictive system

**Figure 3:**
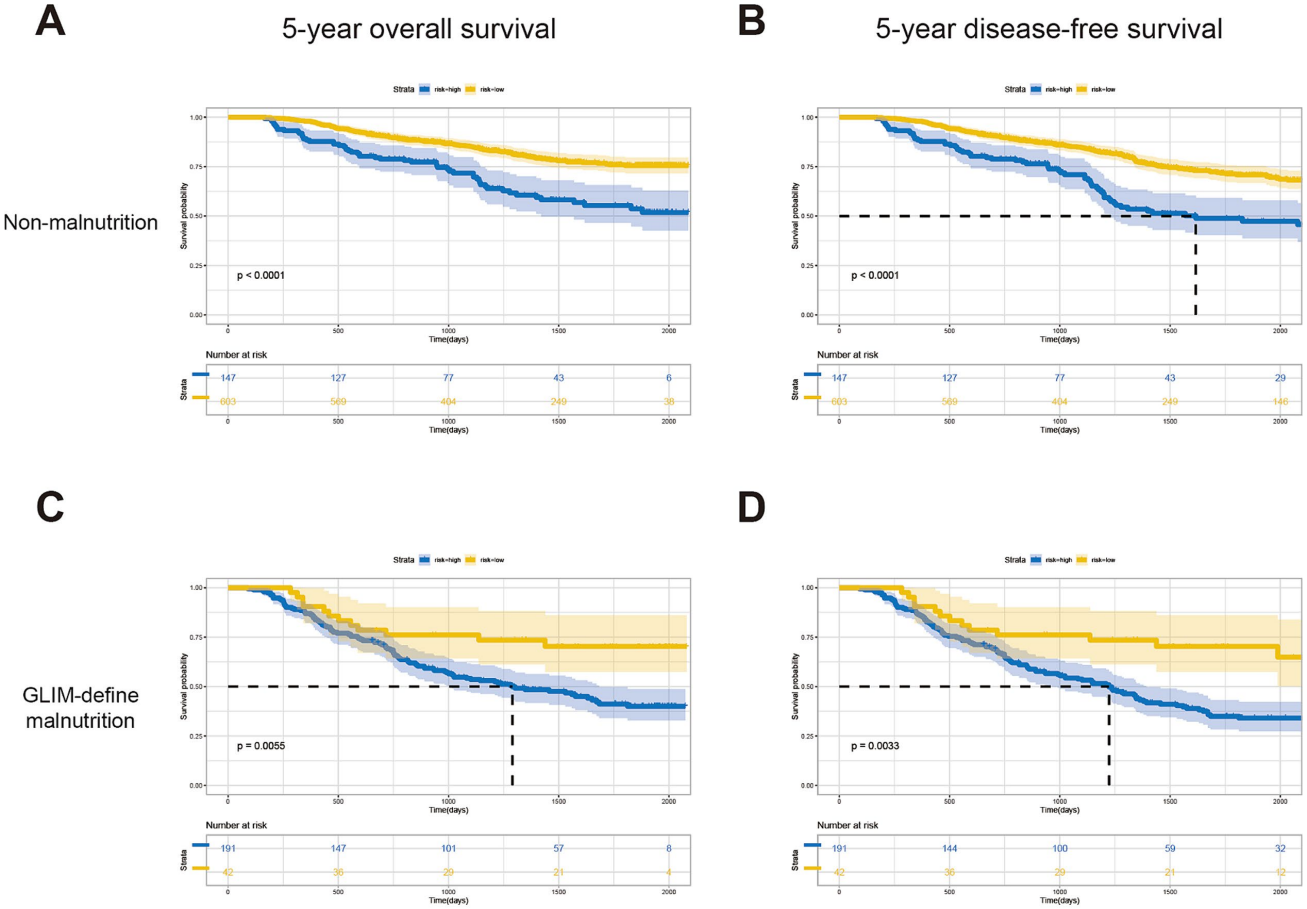
Kaplan–Meier curves for patients in the low-risk and high-risk groups of different subgroups. 5-year OS **(A)** and DFS **(B)** for patients without malnutrition; 5-year OS **(C)** and DFS **(D)** for patients without malnutrition.

Based on this predictive nomogram, the risk score of each individual was calculated, and the patients were divided into a low-risk group (risk score < 194.90) and a high-risk group (risk score ≥ 194.90) according to the cutoff value. As demonstrated in Table 4, significantly more patients were diagnosed with malnutrition by GLIM criteria (57.01 vs. 6.48%, *p* < 0.001) or with nutritional risk by NRS-2002 (64.18 vs. 17.59%, *p* < 0.001) in the high-risk group. More patients with CCI ≥ 2 were also identified in the high-risk group (23.18 vs. 14.04%). Significantly fewer incidences of total complications (19.44 vs. 31.94%, *p* < 0.001) and severe complications (2.78 vs. 5.37%, *p* = 0.040) were found in patients with low risk, which may explain the shorter postoperative hospital stay in the low-risk group (*p* = 0.002). Moreover, the following analysis indicated favorable predictive effects of this scoring system for 5-year OS (*p* < 0.001) and 5-DFS (*p* < 0.001) in subgroups of patients with or without GLIM-defined malnutrition (Figures 3A–D), as well as in patients with the same TNM stage (Supplementary Figure 3). Taken together, these results indicated the favorable prognostic effect in the long-term prognosis of the novel predictive system, which is superior to a single common clinical factor related to nutritional status.

**Table 4:** Comparison of nutritional status and short-term outcomes of training and validation cohorts.

	Low risk (*n* = 648)	High risk (*n* = 335)	*p*-value
GLIM-defined malnutrition, *n* (%)	42 (6.48)	191 (57.01)	<0.001
NRS-2002 ≥ 3, *n* (%)	114 (17.59)	215 (64.18)	<0.001
Charlson comorbidity index, *n* (%)			<0.001
0	339 (52.31)	138 (41.19)	
1	218 (33.64)	116 (34.63)	
≥2	91 (14.04)	81 (23.18)	
Total complications, *n* (%)	126 (19.44)	107 (31.94)	<0.001
Severe complications, *n* (%)	18 (2.78)	18 (5.37)	0.040
Postoperative hospital stay, median (IQR) (d)	11 (5)	13 (6)	0.002
Readmissions within 30 days of discharge, *n* (%)	13 (2.01)	11 (3.28)	0.219

## 4 Discussion

Malnutrition is highly related to the postoperative complications of patients with gastrointestinal cancer who underwent radical surgery, including anastomotic stoma, and severe wound infection, which would delay postoperative recovery and affect longer-term outcomes (22–24). However, it still turned out to be difficult to predict the clinical prognosis of those patients individually according to their nutritional status (25). In our present study, we introduced a novel nomogram based on GLIM criteria and other factors related to cancer staging and nutritional status. ROC and DCA curves indicated the favorable performance of the nomogram in predicting survival for patients with CRC. In addition, further analysis also demonstrated better predictive effectiveness for prognostic prediction compared to the traditional factors, such as serum Hb level, BMI, and NRS-2002.

Malnutrition can be identified in 16.86–47.60% of the patients with gastrointestinal cancer. In advanced phrases, the incidence can be as high as 70% (4, 26–28). Malnutrition is highly related to adverse postoperative events and survival outcomes. However, single indicators such as BMI and serum prealbumin turned out to be insufficient for diagnosing malnutrition or predicting prognosis accurately under varying circumstances (29–31). In addition, assessments of nutritional status fail to achieve consistency for an individual when evaluated using different criteria (2). This may explain the diverse incidences of malnutrition in the same disease and difficulties in predicting clinical outcomes merely based on nutritional status (28). Therefore, it is vital to develop a more individual system to diagnose malnutrition for prognostic prediction by taking other disease-specific factors into account. For GLIM criteria, the capacity of mortality prediction has been recently demonstrated in patients with gastrointestinal cancer in our previous studies (9, 32). Intriguingly, however, a few researchers attempted to combine GLIM with other clinical factors such as body composition and surgical factors to achieve a more precise prediction or assessment, which indicated the potential for improvements in the GLIM criteria (4, 26). Herein, along with GLIM-defined malnutrition, we developed a novel and simple model for prediction, which also contained the significant prognostic factors selected using the Cox regression. This model demonstrated superiority over traditional criteria for assessing malnutrition in terms of prognostic prediction.

In the current study, among all the clinical factors concerning skeletal muscle quantity and quality, handgrip strength, gait speed, and SMI were the independent risk factors for the 5-year OS according to the univariate analysis, but only handgrip strength was identified using multivariate analysis to develop the predictive model. Indicators for skeletal muscle quantity (SMI), function (handgrip strength), and physical performance (gait speed) are usually used to diagnose diseases of muscular disorder, including sarcopenia and cachexia. These measures are indicative of cancer-related malnutrition due to tumor progression and consumption (33, 34). Gait speed was reported to be associated with disable-free survival in healthy older people (35) and serves as a predictor for poorer survival of patients ≥75 years of age with malignancies, such as blood cancer (36). However, as the increasing burden of early-onset CRC (first diagnosis <50 years old) has been noticed in recent years (1, 37) and the mean age of our cohort is only 63.17 years, gait speed might not be a favorable predictor for the survival of CRC patients. Handgrip strength is regarded as a reliable element to evaluate muscle function and the total skeletal muscle mass, for its simple and non-invasive method and shows a relatively high consistency with the SMI, calculated according to CT scanning to represent the muscle mass of the whole body (38). Recent studies also recommended handgrip strength to be the criterion for malnutrition-related disease in advanced cancers such as cachexia (39, 40). Consistent with these results, handgrip strength was identified by multivariate analysis, with a *p*-value of 0.012, to establish a predictive model in our study. Considering that GLIM contains only reduced muscle mass but without handgrip strength in phenotypic criteria, our research suggested that combining parameters of muscle function may help to improve the effect of GLIM in predicting prognosis.

For most cases of advanced cancer, cure still remains elusive; optimal nutritional status endows patients with tolerance to aggressive treatments or recurrence after the failure of long-term anticancer treatment (41). Several studies have mentioned that malnourished patients experience worse survival after surgery, which is shown in our results (Figure 1). Moreover, surprisingly, we noticed that although the long-term outcomes are highly related to the preoperative nutritional status, the AUC value of our predictive model based on GLIM decreases by time (0.729, 0.703, and 0.683, respectively, for predicting 1-year, 3-year, and 5-year OS in the validation cohort). It is reasonable to find that malnourished patients experience a higher incidence of postoperative complications (Table 3), which would affect 1-year survival (42). For the prognosis over 1 year, factors concerning postoperative recovery, including proper supportive care, intake of adequate amounts of food, and even additional oral nutritional supplements after surgery, would improve the prognosis and prolong survival (43, 44). Therefore, preoperative nutritional status demonstrated its effectiveness in predicting short- or mid-term outcomes, which would decline gradually over time in predicting long-term survival for CRC patients. Thus, we think it is necessary to evaluate the nutritional status not only before surgery but also to perform regular assessments postoperatively to provide the best supportive or integrated palliative care in time to improve the long-term survival of patients with CRC.

In conclusion, our studies demonstrated that combining handgrip strength and other factors, including serum albumin and TNM stage, would improve the capacity of GLIM in predicting the long-term survival of patients with CRC. A nomogram was also established based on GLIM-defined malnutrition and screened factors as a predictive model. The limitation of this study includes its single-center nature and exclusion of cases with distant metastasis, as only a minority of these patients received radical surgery. Although with the limitations above, the predictive system in the current study exhibited favorable effects for prognostic prediction in both malnutritional and non-malnutritional patients, respectively, which would merit the individual prediction of clinical outcomes of patients with CRC. Further studies concerning regular assessments of nutritional status after surgery should be performed to improve the prediction of CRC patients.

## Data Availability

The datasets presented in this article are not readily available because the data used and/or analyzed during the current study are available from the corresponding author on reasonable request. Requests to access the datasets should be directed to YP, 13506641535@139.com.
