# Supplementary material for: Development and validation of a novel prognostic prediction system based on GLIM-defined malnutrition for colorectal cancer patients post-radical surgery
Source: Front Nutr. 2024 Oct 22;11:1425317. doi: 10.3389/fnut.2024.1425317 (PMC11536661; doi:10.3389/fnut.2024.1425317)
Supplement: Supplementary file 1 [file Data_Sheet_1.docx]

Supplementary Material

# Supplementary Tables

**Supplementary table1** Numbers of cases with malnutrition meeting each phenotypical criterion of GLIM

| Nutritional characteristic | Phenotypic criteria | | | GLIM-defined malnutrition, n (%) |
| --- | --- | --- | --- | --- |
|  | Weight loss, n (%) | Low BMI, n (%) | Reduced muscle mass, n (%) |  |
| Total, n (%) | 176 (17.90) | 72 (7.32) | 239 (24.31) | 233 (23.70) |
| Training cohort, n (%) | 118 (17.99) | 48 (7.32) | 160 (24.39) | 163 (24.85) |
| Validation cohort, n (%) | 58 (17.74) | 24 (7.34) | 79 (24.16) | 70 (21.41) |

# Supplementary Figures


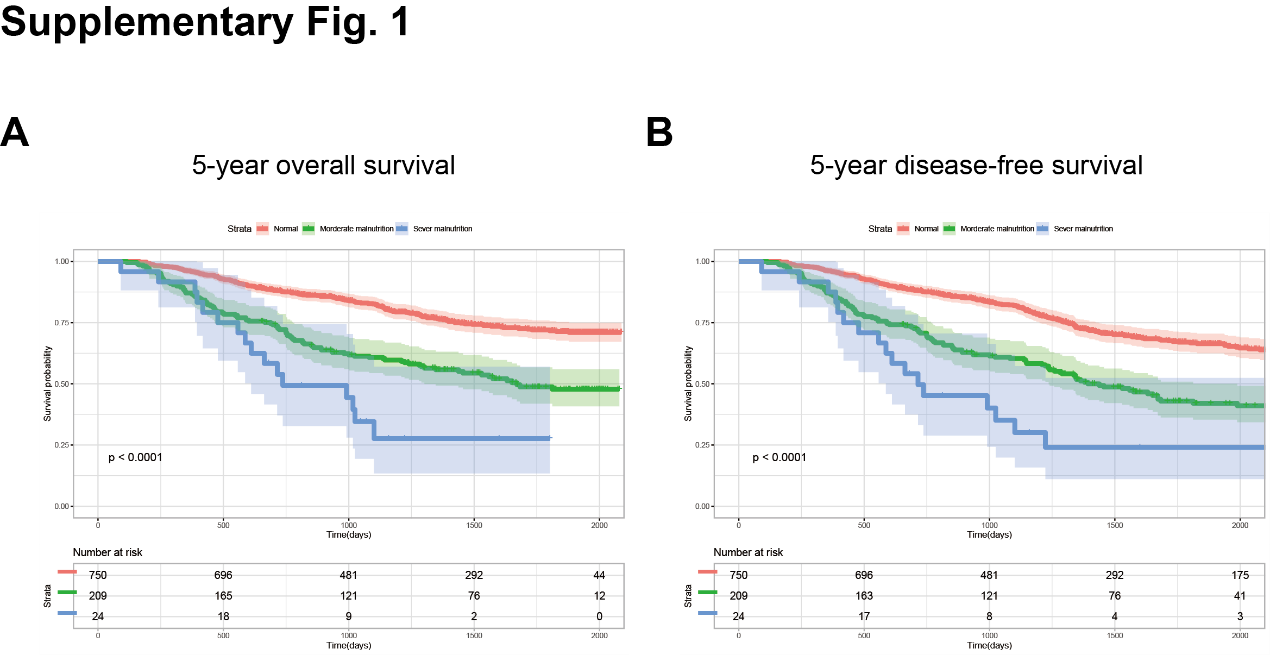


**Supplementary Fig. 1** Kaplan-Meier curves of 5-year OS (A) and 5-year DFS (B) for the patients with different nutritional status defined by GLIM criteria.


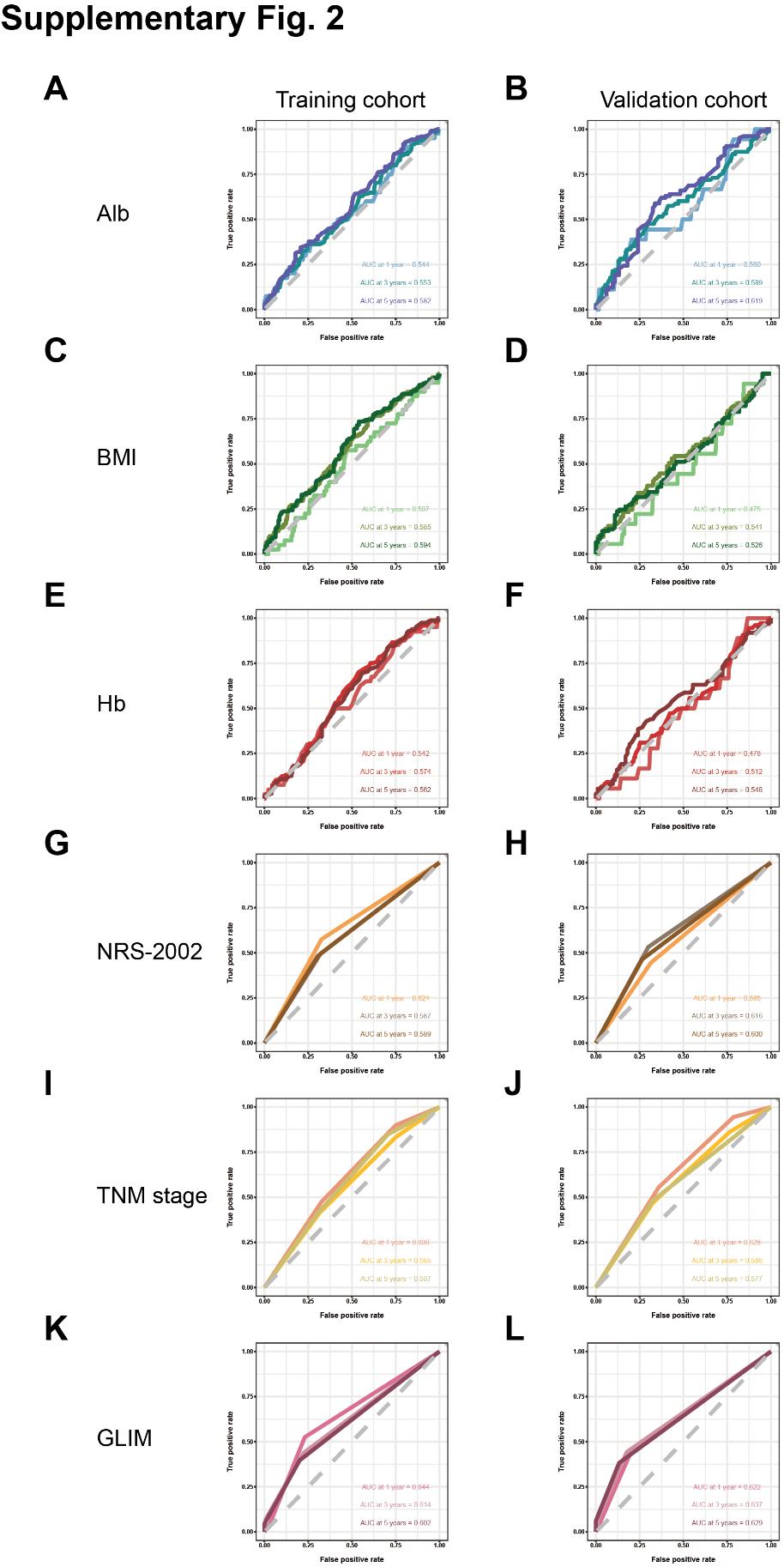


**Supplementary Fig. 2** AUC for survival prediction of Alb (A), BMI (C), Hb (E), NRS-2002 (G), TNM stage (I), and GLIM criteria (K) in training cohort, as well as Alb (B), BMI (D), Hb (F), NRS-2002 (H), TNM stage (J), and GLIM criteria (L) in validation cohort.


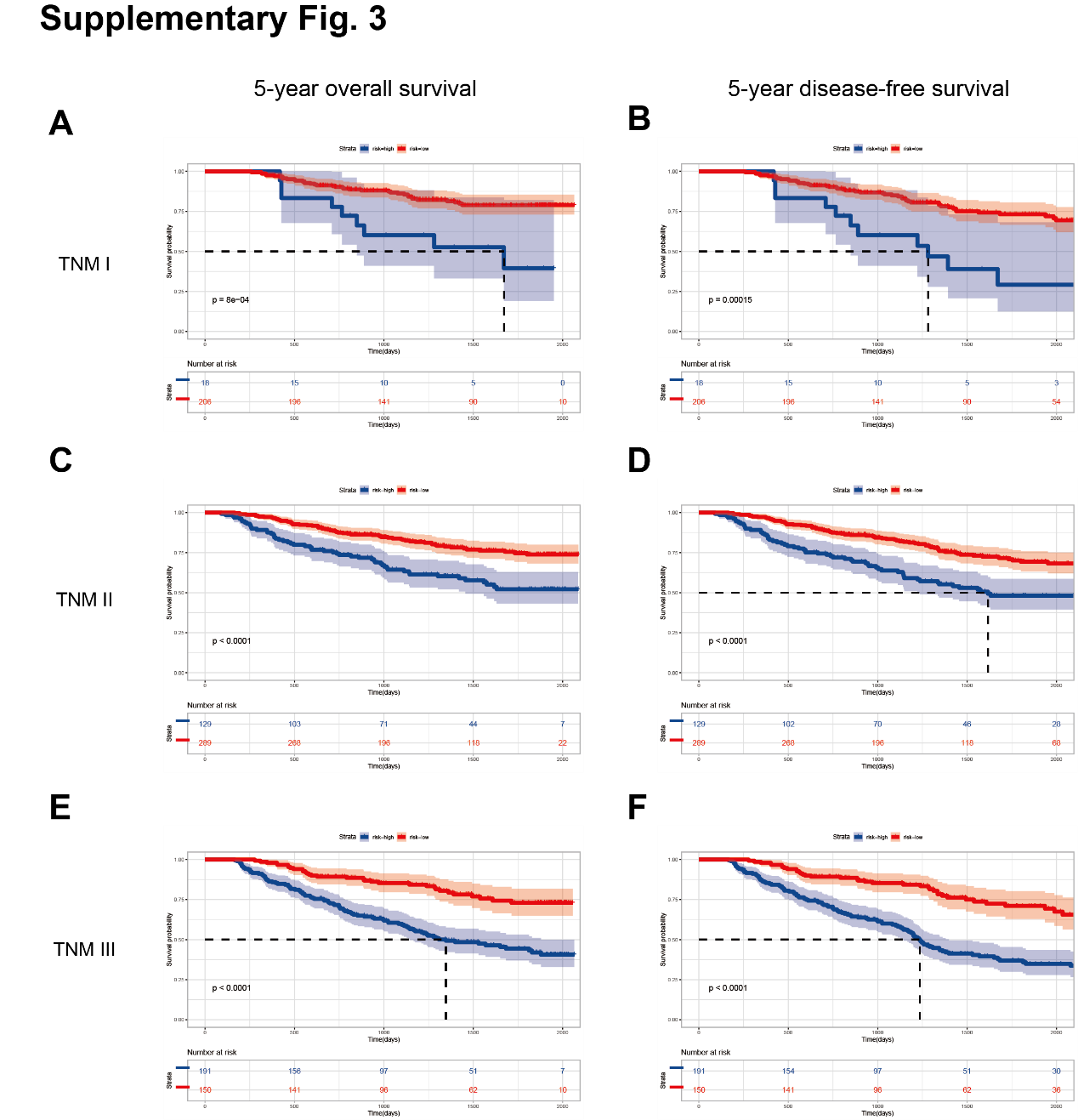


**Supplementary Fig. 3** Kaplan-Meier curves for patients in Low-risk and High-risk group of different subgroups. 5-year OS (A) and DFS (B) for patients with TNM stage I colorectal cancer; 5-year OS (C) and DFS (D) for patients with TNM stage II colorectal cancer; 5-year OS (E) and DFS (F) for patients with TNM stage III colorectal cancer.
